# The Contribution of Age and Weight to Blood Pressure Levels Among Blacks and Whites Receiving Care in Community-Based Primary Care Practices

**DOI:** 10.5888/pcd12.150069

**Published:** 2015-09-24

**Authors:** Athena Wing-ga Kan, Tanvir Hussain, Kathryn A. Carson, Tanjala S. Purnell, Hsin-Chieh Yeh, Michael Albert, Lisa A. Cooper

**Affiliations:** Author Affiliations: Athena Wing-ga Kan, River Hill High School, Clarksville, Maryland; Tanvir Hussain, Tanjala S. Purnell, Michael Albert, Department of Medicine, Johns Hopkins University School of Medicine, Baltimore, Maryland; Kathryn A. Carson, Department of Epidemiology, Johns Hopkins Bloomberg School of Public Health; Hsin-Chieh Yeh, Department of Medicine, Johns Hopkins University School of Medicine, Baltimore, Maryland, and Department of Epidemiology, Johns Hopkins Bloomberg School of Public Health, Baltimore, Maryland. Dr Cooper is affiliated with the Department of Medicine, Johns Hopkins University School of Medicine, Baltimore, Maryland, and the Department of Epidemiology, Johns Hopkins Bloomberg School of Public Health, Baltimore, Maryland.

## Abstract

We examined whether race and age, risk factors for obesity and hypertension, affect the association of obesity with elevated blood pressure (BP). Using electronic medical record data, we conducted a cross-sectional study of adult patients seen at 6 Maryland primary care clinics from September 2011 through June 2012. The risk for higher BP among patients younger than 65 years and in an elevated weight category was greater for both races but was higher for whites than blacks. For patients aged 65 years or older, weight had little impact on systolic BP, suggesting that approaches involving weight loss to address elevated BP should focus on populations younger than 65.

## Objective

Blacks have higher BP levels than whites and are disproportionately overweight compared with whites, increasing their risk for uncontrolled BP (1,2). These disparities persist at older ages, when prevalence of hypertension is highest (3,4). Blacks aged 65 or older have a higher rate of treatment for hypertension but still face poorer BP control than do whites in the same age group (5,6). Few studies examine how race, age, and weight interact to determine risk of increasing BP. This study aims to examine how race and age modify the association of weight with BP.

## Methods

We conducted a cross-sectional study of all adult patients (≥18 years) whose primary care provider (PCP) was located at one of 6 Johns Hopkins Community Physicians (JHCP) sites or who were seen at one site most of the time if they lacked a PCP. We used data from the most recent visit that occurred from September 10, 2011, through June 24, 2012, during which BP was measured and data used to calculate body mass index (BMI) were collected. The clinics serve patients from diverse socioeconomic backgrounds; 4 practices are in underserved areas. Johns Hopkins Medicine institutional review board and JHCP’s clinical research committee approved the study. Patient informed consent was waived.

Clinic staff measured BP with automated BP measurement devices (Omron HEM-907XL), following a standard protocol. This device inflates the BP cuff after a 3-minute rest period and displays the mean of 3 measurements each separated by 30 seconds. We categorized those who reported their race or ethnicity to be Hispanic as Hispanic. For those who did not report ethnicity as Hispanic, we categorized those who reported race as “Caucasian” or “Black or African American” as non-Hispanic white or non-Hispanic black. We categorized those reporting any other race and those with missing race and ethnicity data as “other/unknown.” We calculated age using self-reported birthdate. Height and weight measures from the electronic medical record were used to calculate BMI. We excluded underweight patients (BMI<18.5), a heterogeneous group at greater or lower risk for poor outcomes.

Frequencies and percentages summarized and χ^2^ tests compared demographic characteristics, overall and stratified by BMI category. We used general linear models regression analysis to assess associations of BMI category with systolic and diastolic BP while adjusting for age and diabetes status. We repeated the analysis, stratifying by race, age category, and both race and age. We created an ordinal variable for BMI category to test for trend and assess effect modification of the BMI-BP association with race and age. Analyses were performed by using SAS version 9.3 (SAS Institute, Inc). All *P* values are two-sided.

## Results

We examined the demographic characteristics of the patient sample, overall and by BMI category (Table 1) and the association of BMI category with systolic and diastolic blood pressure levels, adjusted for age and diabetes status (Table 2). Overall, the levels increased with BMI (*P* for trend <.001).

**Table 1:** Characteristics of Patients, Overall and Stratified by BMI Category, Study of Association of Age, Race, and Weight With Blood Pressure, Baltimore, 2011-2012

Characteristic	All Patients	BMI Category, kg/m^2^					*P* Value
		Normal	Overweight	Obese Class I	Obese Class II	Obese Class III	
		18.5–24.9	25.0–29.9	30.0–34.9	35.0–39.9	≥40.0	
		n (%)					
**Sex**							<.001
Female	25,154 (62.1)	6,606 (68.1)	6,743 (54.5)	5,314 (57.9)	3,315 (66.4)	3,176 (74.8)	
Male	15,339 (37.9)	3,099 (31.9)	5,627 (45.5)	3,862 (42.1)	1,678 (33.6)	1,073 (25.2)	
**Race/Ethnicity^a^**							
Hispanic	951 (2.4)	248 (2.6)	334 (2.7)	216 (2.4)	99 (2.0)	54 (1.3)	<.001
Non-Hispanic black	16,605 (41.0)	2,802 (28.9)	4,544 (36.7)	4,206 (45.8)	2,580 (51.7)	2,473 (58.2)	
Non-Hispanic white	20,908 (51.6)	5,835 (60.1)	6,796 (54.9)	4,449 (48.5)	2,185 (43.8)	1,643 (38.7)	
Other/unknown	2,029 (5.0)	820 (8.4)	696 (5.6)	305 (3.3)	129 (2.6)	79 (1.9)	
**Age**							
<65 years	32,742 (80.9)	7,838 (80.8)	9,526 (77.0)	7,400 (80.7)	4,211 (84.3)	3,767 (88.7)	<.001
≥65 years	7,751 (19.1)	1,867 (19.2)	2,844 (23.0)	1,776 (19.4)	782 (15.7)	482 (11.3)	
**Insurance type^b^**							
Medicaid	4,168 (10.3)	910 (9.4)	968 (7.8)	909 (9.9)	633 (12.7)	748 (17.6)	<.001
Private	28,322 (69.9)	6,951 (71.6)	8,726 (70.5)	6,460 (70.4)	3,433 (68.8)	2,752 (64.8)	
Medicare	6,786 (16.8)	1,569 (16.2)	2,258 (18.2)	1,524 (16.6)	785 (15.7)	650 (15.3)	
Uninsured	775 (1.9)	159 (1.6)	287 (2.3)	178 (1.9)	99 (2.0)	52 (1.2)	
Unknown	442 (1.1)	116 (1.2)	131 (1.1)	105 (1.1)	43 (0.9)	47 (1.1)	
**Diabetes**							
Yes	6,135 (15.2)	588 (6.1)	1,489 (12.0)	1,685 (18.4)	1,199 (24.0)	1,174 (27.6)	<.001
No	34,358 (84.8)	9,117 (93.9)	10,881 (88.0)	7,491 (81.6)	3,794 (76.0)	3,075 (72.4)	

Abbreviation: BMI, body mass index.

a Before 2011, the electronic medical record did not include a field for ethnicity. Those who were recorded as Hispanic race or Hispanic ethnicity were coded here as Hispanic. Those whose race was recorded as “Caucasian” and “Black or African American” and did not report ethnicity as Hispanic were categorized as non-Hispanic white and non-Hispanic black.

b Insurance type was categorized as Medicaid if patient had any Medicaid insurance, then private if patient had any private insurance, then Medicare if patient had Medicare, then uninsured if coded as self-pay. If none of these were coded, then insurance type was categorized as unknown.

**Table 2:** Association of Body Mass Index With Systolic and Diastolic Blood Pressure^a^, Study of Association of Age, Race, and Weight With Blood Pressure, Baltimore, 2011-2012

Patients	BMI Category, kg/m^2^					*P* for Trend
	Normal	Overweight	Obese Class I	Obese Class II	Obese Class III	
	18.5–24.9	25.0–29.9	30.0–34.9	35.0–39.9	≥40.0	
**Systolic BP, mm Hg**						
All patients	122.6 (0.19)	124.9 (0.17)	125.9 (0.18)	126.6 (0.23)	126.6 (0.25)	<.001
Age <65 years	120.5 (0.22)	123.4 (0.20)	124.3 (0.21)	125.3 (0.25)	125.1 (0.26)	<.001
Age ≥65 years	131.7 (0.46)	131.8 (0.36)	133.0 (0.44)	132.4 (0.66)	132.8 (0.84)	.07
Non-Hispanic white	121.9 (0.25)	124.3 (0.23)	125.0 (0.26)	125.4 (0.34)	125.7 (0.38)	<.001
Non-Hispanic black	124.9 (0.35)	126.1 (0.28)	127.0 (0.28)	127.7 (0.34)	127.4 (0.35)	<.001
**Age <65 years**						
Non-Hispanic white	119.4 (0.29)	122.7 (0.28)	123.4 (0.30)	124.4 (0.37)	124.2 (0.40)	<.001
Non-Hispanic black	123.2 (0.39)	124.7 (0.32)	125.4 (0.31)	126.0 (0.37)	125.9 (0.36)	<.001
**Age ≥65 years**						
Non-Hispanic white	130.3 (0.57)	130.6 (0.46)	131.4 (0.57)	129.7 (0.84)	131.8 (1.14)	.40
Non-Hispanic black	134.0 (0.83)	133.6 (0.62)	135.2 (0.72)	136.4 (1.08)	134.1 (1.27)	.16
**Diastolic BP, mm Hg**						
All patients	69.1 (0.13)	72.7 (0.12)	75.5 (0.13)	77.3 (0.16)	78.5 (0.17)	<.001
Age <65 years	70.4 (0.15)	74.0 (0.14)	76.7 (0.15)	78.5 (0.18)	79.5 (0.18)	<.001
Age ≥65 years	67.5 (0.27)	68.3 (0.22)	69.8 (0.26)	70.3 (0.40)	71.0 (0.51)	<.001
Non-Hispanic white	67.7 (0.17)	71.4 (0.16)	74.3 (0.18)	75.8 (0.23)	77.2 (0.26)	<.001
Non-Hispanic black	71.5 (0.24)	74.1 (0.19)	76.4 (0.20)	78.4 (0.24)	79.3 (0.24)	<.001
**Age <65 years**						
Non-Hispanic white	69.0 (0.20)	73.0 (0.19)	75.6 (0.21)	77.5 (0.26)	78.4 (0.28)	<.001
Non-Hispanic black	73.1 (0.28)	75.3 (0.22)	77.5 (0.22)	79.2 (0.26)	80.3 (0.26)	<.001
**Age ≥65 years**						
Non-Hispanic white	66.5 (0.35)	67.2 (0.28)	68.9 (0.35)	68.4 (0.52)	70.2 (0.70)	<.001
Non-Hispanic black	69.0 (0.48)	69.9 (0.37)	70.8 (0.42)	72.8 (0.63)	72.1 (0.74)	<.001

Abbreviations: BMI, body mass index; BP, blood pressure.

a Results presented are least squares means and standard error of the means adjusted for age (in years) and diabetes.

Among patients younger than 65, the association of BMI with blood pressure (both systolic and diastolic) persisted (*P* for trend <.001). Among patients aged 65 or older, the association of BMI with diastolic BP, but not systolic BP, was significant. The interaction of age and BMI was significant (*P* < .001).

The association of BMI with both systolic and diastolic BP was positive among non-Hispanic blacks and whites. The interaction of BMI and race was significant (*P* < .001), demonstrating that the association of BMI and BP was greater for whites.

Among patients younger than 65, the association of BMI with systolic and diastolic BP was positive among non-Hispanic blacks and whites (*P* for trend <.001 for both groups), and the interaction between BMI and race was significant (*P* < .001). Among patients 65 years or older, the associations of BMI with diastolic BP were consistently positive for non-Hispanic blacks and whites, whereas the associations of BMI with systolic BP were consistently nonsignificant. The interaction of race and BMI was not significant for this age group.

## Discussion

The association of BMI with systolic BP was positive and significant among patients younger than 65, stronger among whites than blacks, and not observed among patients 65 or older. The association of BMI with diastolic BP was positive and significant among patients of all ages and among both blacks and whites.

One potential explanation for the weaker association of weight with systolic BP at older ages is that obesity-related arterial stiffening may occur faster at younger ages, slowing as people age. Another explanation may be healthy survival bias — overweight people who survive beyond age 65 may be as healthy as their normal-weight counterparts. Another explanation may be greater use of health care through Medicare for blacks and whites aged 65 or older, which may increase access to care for obesity-related conditions (7,8).

One limitation of this study is that the data are cross-sectional. Causal inferences cannot be made; obesity may have led to higher BP or higher BP may have led to increased BMI. The latter is unlikely because high BP is typically asymptomatic and has little effect on other risk factors for obesity. Second, this sample may not be generalizable. National Health and Nutrition Examination Survey data show that our study population has a higher percentage of women, black people, people younger than 65, and non-Hispanic people than is in the United States population (9). Our study population was also in a health care system, where patients receive care for hypertension and other conditions. Third, we lacked medication adherence data, which may differ by age and race.

This study suggests that weight loss approaches to reduce systolic BP should focus on black and white populations younger than 65. Approaches targeting older populations may need to focus on other factors, such as health system barriers or broader social determinants of health. However, weight loss interventions may lower the diastolic BP of blacks and whites, regardless of age.
